# Treatment of pubic symphysis infection with vancomycin-loaded calcium sulfate artificial bone combined with debridement in pediatric patients: a case report and literature review

**DOI:** 10.3389/fsurg.2025.1619126

**Published:** 2025-10-21

**Authors:** Su De-zhi, Wu Gang-quan, Ye Shi-min, Wang Kun, Lu Ying-chou, Zhai Guo-min, Zhu Zhe-min, Yang Yi, Zhuo Bao-biao

**Affiliations:** Department of Pediatric Surgery, Dongguan Maternal and Child Health Hospital, Dongguan, Guangdong, China

**Keywords:** pubic symphysis infection, pubic osteomyelitis, pediatric, absorbable bone graft, vancomycin-loaded calcium sulfate

## Abstract

**Objective:**

To investigate the clinical characteristics of pubic symphysis infection and evaluate the safety and efficacy of vancomycin-loaded calcium sulfate (VCS) artificial bone combined with debridement in treating these infections in adolescents.

**Methods:**

We retrospectively analyzed a clinical case and supplemented our findings with a narrative literature review. The evaluation focused on a 13-year-old female patient diagnosed and treated at our hospital in February 2025. To contextualize our case, we searched PubMed, Wanfang, China National Knowledge Infrastructure (CNKI), VIP, and China Biology Medicine (CBM) databases for articles available from the establishment of each database up to April 2023. Data from the present case and previously documented cases of pubic symphysis infection in pediatric patients who met the inclusion criteria were compiled. The data, with a particular focus on clinical presentations, treatment modalities, and follow-up outcomes, were analyzed to draw comparative insights regarding treatment efficacy.

**Results:**

A comprehensive literature review identified eight documented pediatric cases of pubic symphysis infection, each presented as individual case reports. Notably, one of these cases lacked identifiable risk factors. All patients were effectively managed through conventional surgical débridement in conjunction with antibiotic therapy. In the current case, the patient underwent a two-stage intervention that included the implantation of vancomycin-loaded calcium sulfate (VCS) beads, débridement of the pubic symphysis, and bilateral pubic fenestration and drainage. This was succeeded by a six-week regimen of postoperative antibiotic therapy. The patient exhibited an excellent recovery, characterized by normalized bilateral hip range of motion and absence of tenderness over the pubic symphysis. Follow-up imaging revealed the resorption of the VCS beads and bilateral callus formation. Unlike previously reported cases, the treatment strategy implemented in this instance obviated the necessity for repeated débridement and dressing changes, thereby significantly reducing patient discomfort.

**Conclusion:**

The application of VCS combined with debridement was effective in controlling infection and promoting bone regeneration in this pediatric case, offering a promising therapeutic approach for similar rare infections.

## Introduction

Pubic symphysis infection is a rare and clinically complex condition, particularly in pediatric and adolescent populations. The symptoms include fever, localized tenderness over the pubic symphysis, and pain exacerbated by hip motion, with potential radiation to the lower abdomen or genital region. Due to its nonspecific clinical presentation, this condition is frequently misdiagnosed, resulting in delayed treatment (1). To date, fewer than 10 pediatric cases have been reported in the Chinese and English literature. In this report, we describe the case of an adolescent patient who was treated with vancomycin-loaded calcium sulfate (VCS) implantation combined with surgical debridement. A review of the literature on published cases in children and adolescents is also provided to investigate the clinical characteristics of this condition and evaluate the efficacy of VCS as an adjunct to debridement in the management of pubic symphysis infections.

## Methods

We reviewed the patient's clinical records, imaging findings, bacteriological data, and postoperative recovery, and conducted a comprehensive literature search.Based on established search strategies from prior studies (2), a comprehensive literature review of pediatric and adolescent pubic symphysis infections was conducted. This involved the searching of the PubMed, Wanfang, China National Knowledge Infrastructure (CNKI), VIP, and China Biology Medicine (CBM) databases for articles published from database inception to April 2025. The English search terms included “osteomyelitis pubic symphysis”, “septic arthritis pubic symphysis”, “pubic symphysitis”, “pubic osteitis”, and “pubic infection”, while Chinese keywords were “耻骨联合感染” (pubic symphysis infection), “耻骨联合化脓性感染” (suppurative pubic symphysis infection), “耻骨联合炎症” (pubic symphysis inflammation), “耻骨骨髓炎” (pubic osteomyelitis), “耻骨感染” (pubic infection), and “耻骨炎症” (pubic inflammation). The criteria for article inclusion in the analysis were: (1) patients aged ≤18 years; (2) confirmed diagnosis of pubic symphysis infection. The exclusion criteria were: (1) cases with ambiguous diagnoses; (2) studies lacking sufficient clinical data for analysis.

## Case introduction

**Figure 1:**
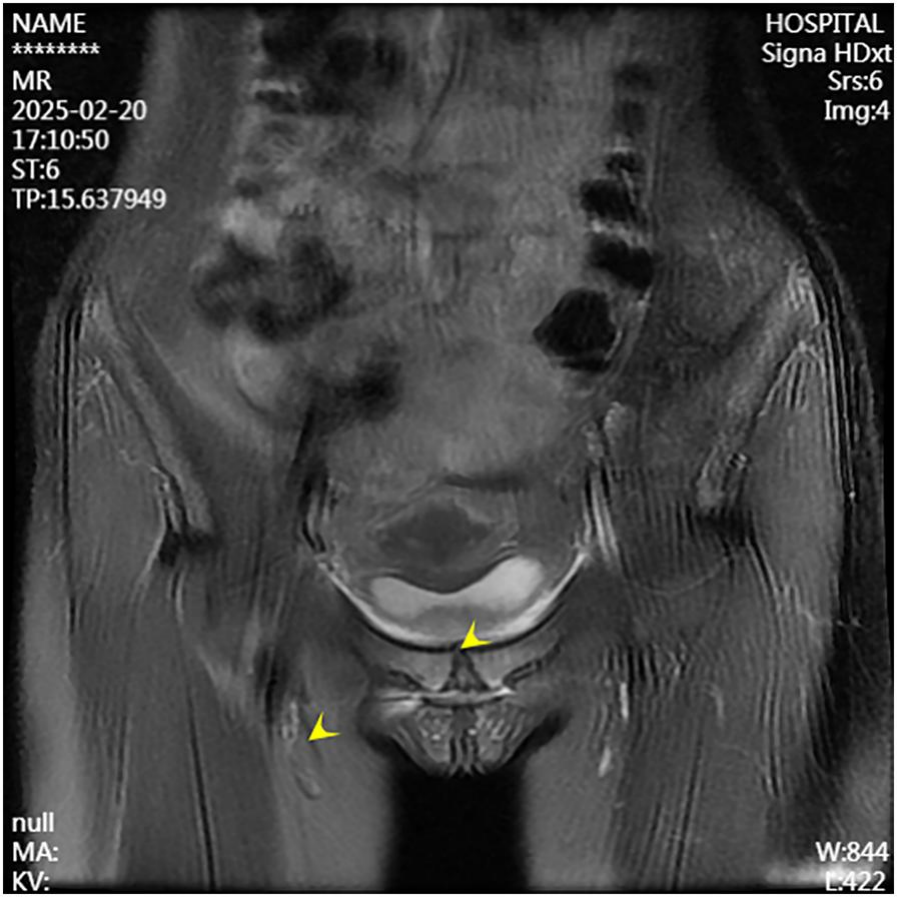
Preoperative MRI indicates infection of the pubic symphysis.

**Figure 2:**
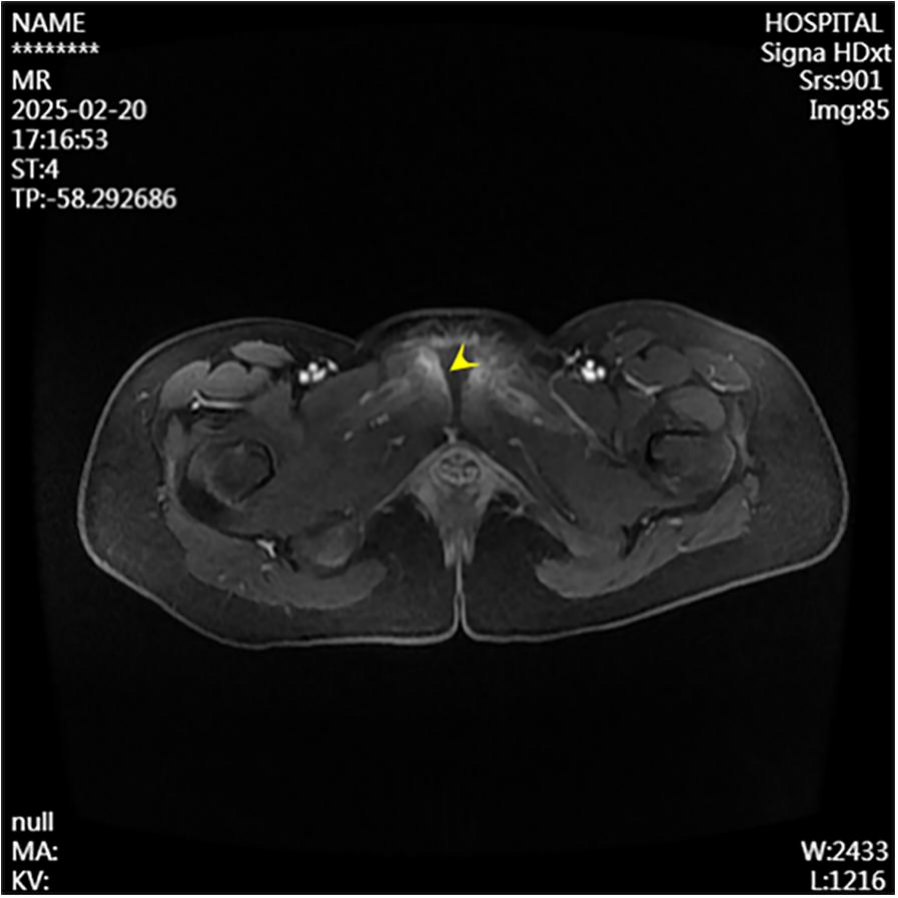
Preoperative MRI shows infection at the pubic symphysis.

A 13-year-old female presented to the Department of Pediatrics at Dongguan Maternal and Child Health Hospital with a 10-day history of intermittent fever and pain in the pubic symphysis region. The patient denied a history of pelvic surgery, local trauma, or strenuous exercise. Her past medical and family histories were unremarkable. Between February 10 and 18, 2025, she was hospitalized at an external institution due to pubic symphysis pain, restricted lower limb mobility, and high fever. Blood cultures identified the presence of methicillin-resistant *Staphylococcus aureus* (MRSA) and she was treated initially with cephalosporins and penicillins which resolved the fever but only partially alleviated the pubic pain. Upon transfer to our hospital, physical examination revealed stable vital signs, no fever, localized tenderness and swelling over the pubic symphysis without erythema or fluctuance, bilateral restricted hip motion (aggravated by adduction and abduction), and an antalgic gait. The laboratory findings included a white blood cell count of 6.08 × 10^9^/L (neutrophils: 64.0%), procalcitonin <0.1 ng/ml, and an erythrocyte sedimentation rate (ESR) of 80 mm/h. Blood cultures confirmed the presence of MRSA. Although intravenous antibiotics had resolved the fever, pubic pain and mobility limitations persisted, particularly with limited hip mobility, notably painful external rotation and adduction, where external rotation was less than 15 degrees and adduction was less than 10 degrees. Consultation with orthopedic specialists led to the suspicion of pubic symphysis infection, prompting an MRI examination. The imaging revealed infection of the pubic symphysis with bilateral osteomyelitis and a small abscess in the posteroinferior muscle layer (Figures 1, 2). Based on these clinical manifestations, laboratory results, and imaging findings, the diagnosis was confirmed as pubic symphysis infection with bilateral osteomyelitis and abscess formation.

This study was approved by the Ethics Committee of Dongguan Maternal and Child Health Hospital, and written informed consent was obtained from the patient's legal guardian.

**Figure 3:**
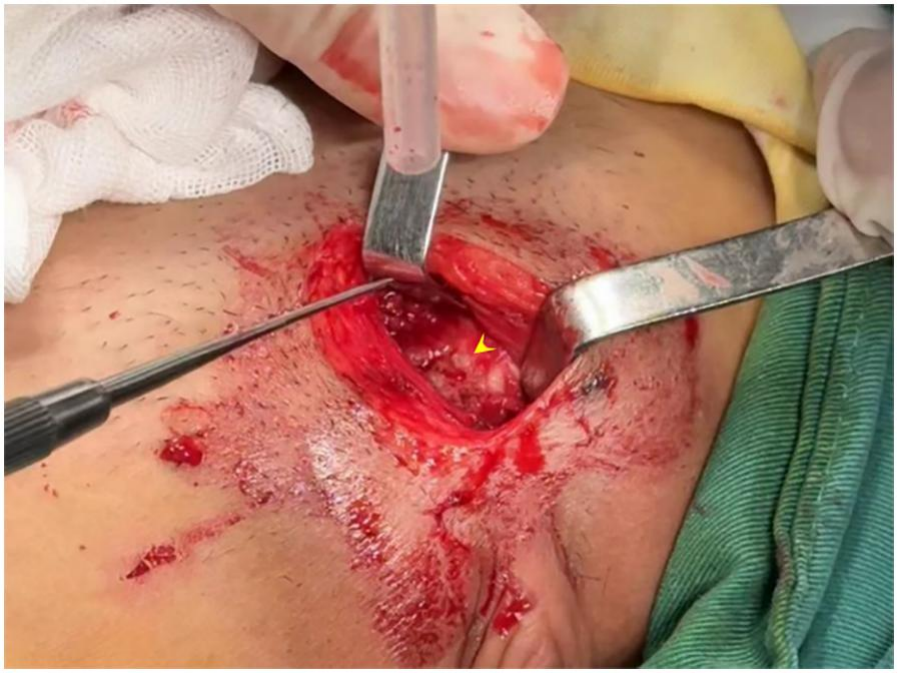
Intraoperative view: fibrous tissue of the pubic symphysis with purulent changes, and pus observed within the medullary cavities of bilateral superior pubic rami.

Following definitive diagnosis, surgical intervention comprising debridement of the pubic symphysis and bilateral pubic bone fenestration with drainage was performed. Intraoperative findings indicated suppurative changes in the fibrocartilaginous tissue of the pubic symphysis, with the identification of purulent material within the medullary cavities of the bilateral superior pubic rami (Figure 3). The necrotic debris attached to the fibrocartilaginous plate of the pubic symphysis and the pus within the superior pubic rami were meticulously debrided, followed by decompression of the affected regions. Histopathological examination indicated granulocyte infiltration within the fibrocartilage of the pubic symphysis and trabecular bone marrow of the pubic rami. MRSA was identified in intraoperative cultures.

**Figure 4:**
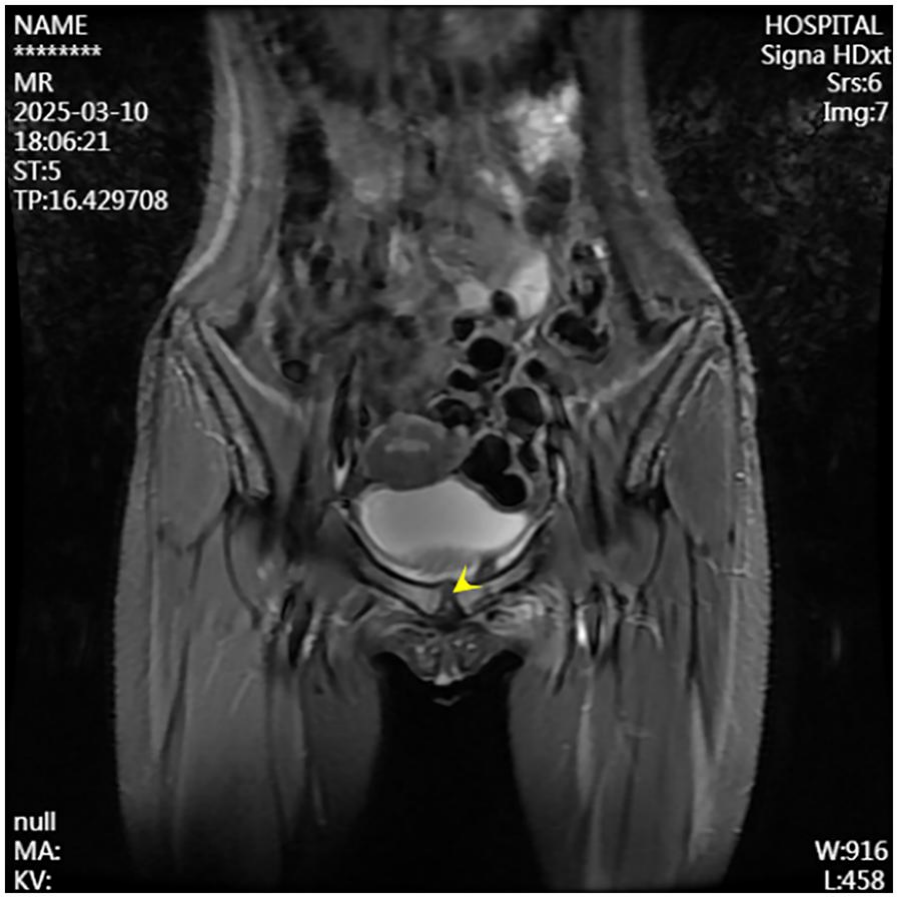
Follow-up MRI at two weeks postoperatively shows controlled infection at the pubic symphysis.

**Figure 5:**
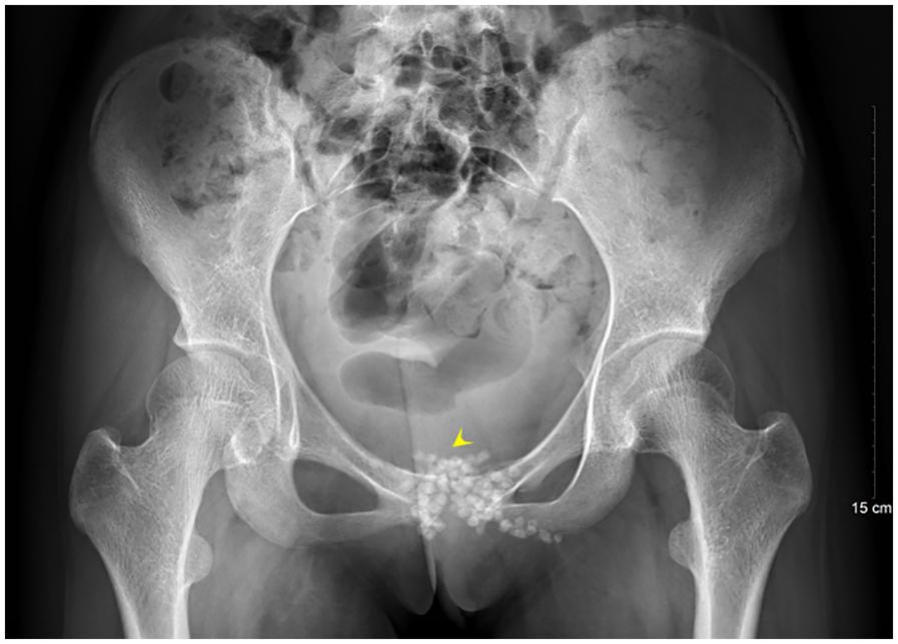
Follow-up pelvic radiograph at two weeks postoperatively reveals the bone defect filled with VCS.

**Figure 6:**
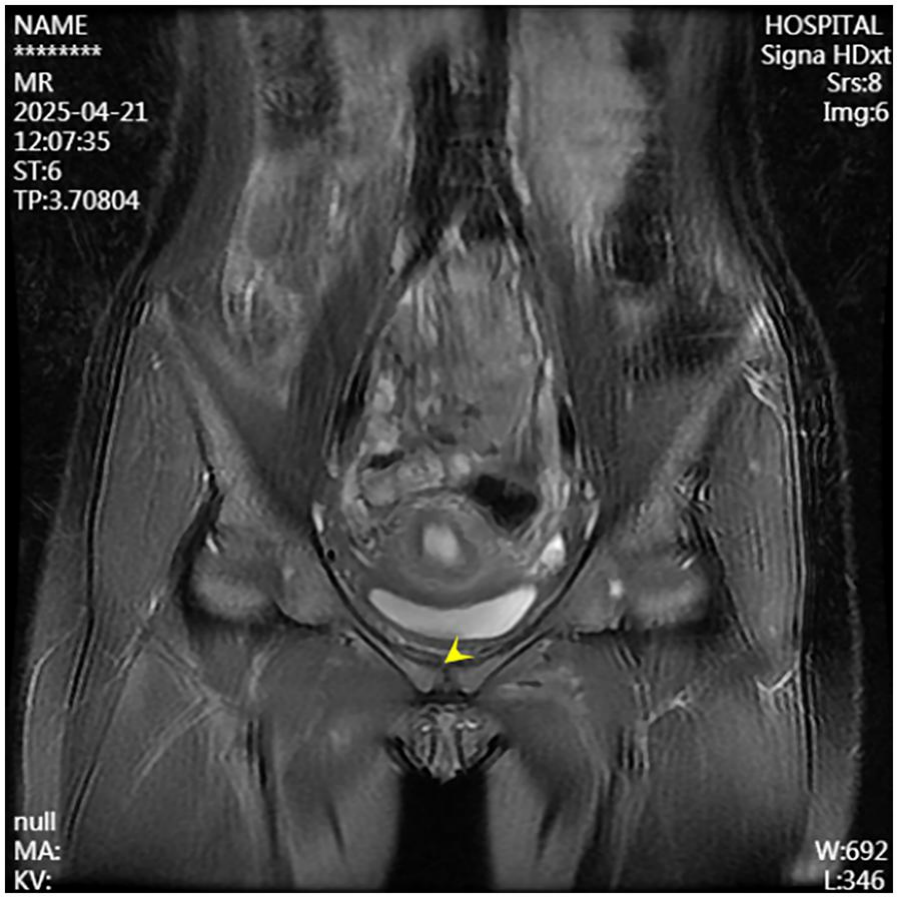
Follow-up MRI at six weeks postoperatively demonstrates resolved infection at the pubic symphysis.

**Figure 7:**
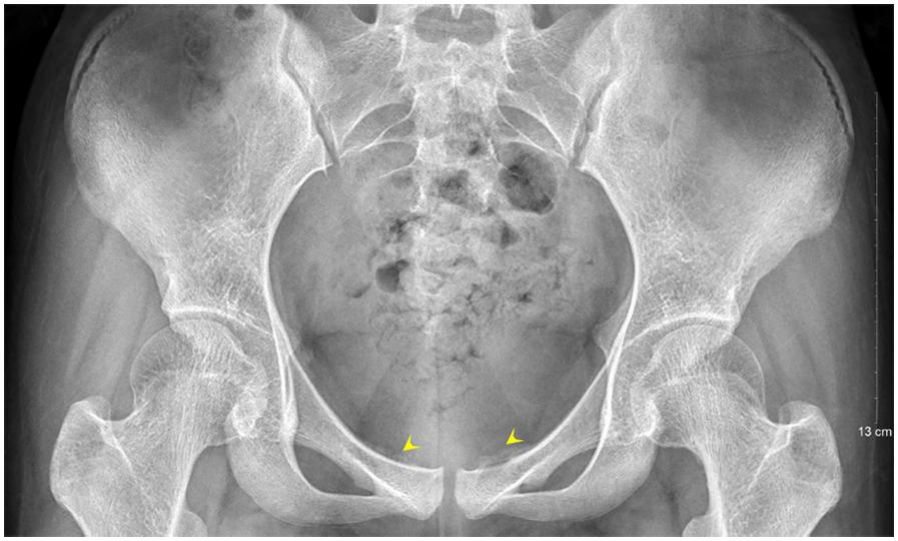
Follow-up pelvic radiograph at six weeks postoperatively shows absorption of the artificial bone and noticeable periosteal reaction.

**Figure 8:**
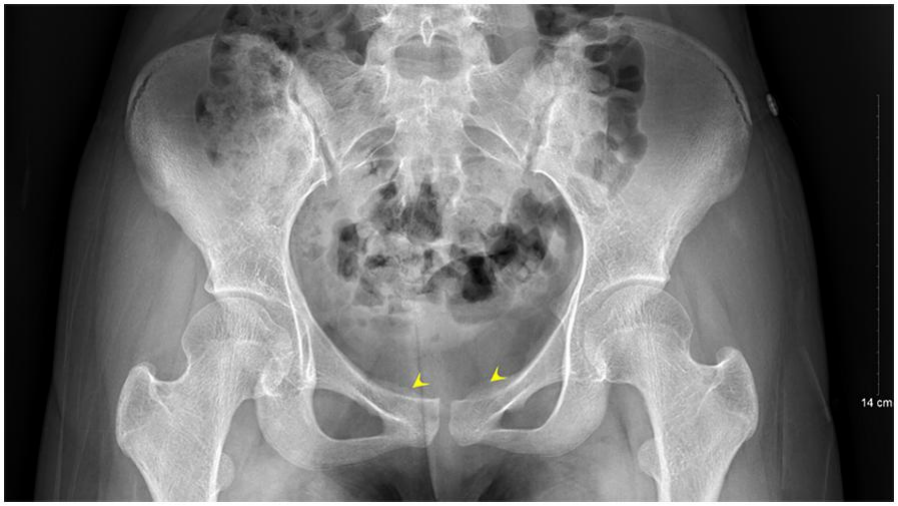
Follow-up pelvic radiograph at 22 weeks postoperatively shows callus formation at the bilateral pubic rami.

A second surgical procedure was performed four days later. This involved VCS implantation, repeat debridement of the pubic symphysis, and bilateral pubic bone fenestration with drainage. During the procedure, 2 grams of vancomycin powder were combined with 5 milliliters of Stimula® calcium sulfate powder and 2.6 milliliters of sterile water for injection. Following approximately 30 s of thorough mixing, a homogeneous paste was achieved. This paste was subsequently placed into a mold to form hemispherical beads with a diameter of 4.0 millimeters. The antibiotic beads were allowed to set for approximately 15 min before being extracted from the mold for intraoperative application. Under fluoroscopic guidance, the beads were implanted into the medullary cavity. Once the cavity was confirmed to be adequately filled, additional VCS beads were applied to cover the cortical surface of the bone window and the defect area, thereby restoring the structural contour of the local bone. Postoperative serum vancomycin levels were assessed every three days starting from the first postoperative day, with dosage adjustments made based on pharmacokinetic analysis to ensure concentrations remained within the target therapeutic range. Concurrently, routine evaluations of hepatic and renal function, electrolytes (including calcium), and superficial ultrasound examinations of the surgical site were conducted, which revealed no significant fluid accumulation. Laboratory results were within normal limits: white blood cell count at 6.01 × 10^9^/L, neutrophil percentage at 54.3%, C-reactive protein at 2.43 mg/L, procalcitonin levels below 0.1 ng/ml, erythrocyte sedimentation rate at 10 mm/h, negative blood cultures, and normal hepatic and renal function, electrolytes, and calcium levels. The patient had no relevant postoperative symptoms such as frequent urination, urgent urination, hematuria, or dysuria.By one week postoperatively, the surgical incision had healed by primary intention, pain at the pubic symphysis had fully resolved, and lower limb motor function had returned to normal, with full hip abduction and internal rotation. Imaging conducted at two weeks post-surgery revealed the presence of residual antibiotic beads and localized infection with osteolytic defects in the pubic symphysis (Figures 4, 5). Following discharge, the patient underwent a four-week regimen of oral vancomycin aimed at eradicating any residual infection and was advised to refrain from engaging in strenuous activities for three months. Subsequent imaging at the six-week mark revealed complete absorption of the calcium sulfate artificial bone, resolution of prior infectious indicators, and a visible periosteal reaction indicative of active bone remodeling (Figures 6, 7). By the 22-week follow-up, callus formation was evident in the bilateral pubic rami, signifying successful osseous healing (Figure 8).

After the exclusion of studies on infections not associated with the pubic symphysis, 9 articles on pediatric infections of the pubic symphysis were identified. One of these was excluded due to inaccessible data from an outdated publication, resulting in 8 eligible case reports published in English (3). Of these, Lorenzo et al. (4) described a 12-year-old child with fever (39–40°C), abnormal gait, and left groin pain; pelvic computed tomography (CT) indicated a pubic symphysis-adjacent abscess, with *Staphylococcus aureus* identified on aspiration culture. The case was managed by surgical debridement and four weeks of antibiotics, resulting in the complete resolution of symptoms by six months (4) Hartshorn et al. (5) reported an 11-year-old with bilateral groin pain, difficulty in walking, and nocturnal enuresis. The child was diagnosed via MRI (pubic symphysis fluid) at four weeks and was treated with intravenous flucloxacillin/cefuroxime for two weeks, followed by oral antibiotics for four weeks (5). Ming-Yen Tsai et al. (6) documented an 8-year-old with bilateral thigh pain and fever; MRI at two days showed the presence of pubic symphysis fluid, osteolysis, and soft tissue edema. The patient was treated with intravenous oxacillin and oral rifampin/dicloxacillin for 39 days (6). Imane El Mezouar et al. (1) described the case of a 16-year-old with fever, pubic/genital pain, and limping; MRI confirmed edema of the pubic symphysis/rectus abdominis, and cultures indicated the presence of methicillin-sensitive *S. aureus* (MSSA). The patient was managed with six weeks of intravenous and four weeks of oral flucloxacillin (1) Alqahtani et al. (7). described a 17-year-old with groin pain, chills, and fever; MRI at three weeks revealed pubic osteomyelitis and thigh abscesses (Group A *Streptococcus*), which was treated surgically followed by six weeks of intravenous antibiotics (7). A 16-year-old patient was described by Smits et al. (8) as experiencing hip/groin pain and fever; MRI at five days showed para-pubic edema/abscess (*S. aureus* on blood culture), and the patient was treated with surgical/image-guided drainage and six weeks of antibiotics (8) Upadhyayula (9). presented the case of a 14-year-old with suprapubic pain/fever; MR enterography demonstrated septic arthritis of the pubic symphysis with cellulitis/abscess caused by MSSA, which was treated by surgical drainage, antibiotics (six weeks intravenous and 14 weeks oral), and steroids for five months (9) Kim et al. (10). described the case of a 16-year-old who did not respond to six weeks of conservative antibiotic treatment, requiring surgery after 16 weeks of therapy, as described in Table 1 (10).

**Table 1:** Clinical characteristics and treatment outcomes of the included studies.

Year	Age/sex	Clinical presentation	Risk factors	Imaging features (MRI/CT)	Microbiological evidence	Treatment strategy	Antibiotic duration	Inflammatory markers
2000	12y/F	Left groin pain, limping, high fever (39 °C–40 °C)	Ballet training	Prevesical abscess involving pubic symphysis and muscles	Blood/pus culture: S. aureus (MSSA)	Surgical debridement + Cloxacillin	4 weeks (2 weeks IV, 2 weeks PO)	ESR: 75 mm/h
2009	11y/M	Bilateral groin pain, limping, no fever	Soccer	Pubic symphysis fluid collection, surrounding muscle edema	Blood culture: Negative	Flucloxacillin	6 weeks (2 weeks IV, 4 weeks PO)	ESR: 65 mm/h
2012	8y/M	Bilateral thigh pain, high fever	No clear risk factors	Pubic symphysis fluid collection, subperiosteal bone destruction	Blood culture: Negative	Penicillin + Dicloxacillin	39 days	ESR: 104 mm/h
2014	16y/M	Pubic pain, fever	Soccer	Pubic symphysis bone edema, surrounding muscle edema	Pus culture: S. aureus (MSSA)	Flucloxacillin	6 weeks	ESR: 25 mm/h
2014	17y/M	Groin pain, fever	Juvenile idiopathic arthritis	Bilateral adductor abscesses, pubic osteomyelitis	Pus culture: Group A Streptococcus	Surgical debridement + antibiotics	6 weeks	ESR: 57 mm/h
2016	16y/F	Right hip/groin pain, fever	Rock climbing	Retro-pubic abscess, bone marrow edema	Blood culture: S. aureus (MSSA)	Surgical drainage + antibiotics	6 weeks	CRP: 150 mg/L
2020	14y/F	Right lower quadrant pain, fever	Track and field	Pubic symphysis fluid collection, bone marrow edema	Pus culture: S. aureus (MSSA)	Surgical debridement + long-term antibiotics	5 months (6 weeks IV, 14 weeks PO)	CRP: 29.8 mg/dl
2023	16y/M	Right hip pain, fever	Soccer	Retro-pubic abscess, bone marrow edema	Blood culture: S. aureus (MSSA)	Surgical drainage + antibiotics	16 weeks	27 mm/h

Y, years old; M, male; F, female; MSSA, methicillin-susceptible Staphylococcus aureus; ESR, erythrocyte sedimentation rate; CRP, C-reactive protein.

## Discussion

The pubic symphysis functions as a crucial stabilizing structure in the anterior pelvis, with its fibrocartilaginous disc and ligamentous complex playing essential roles in both stress transmission and energy absorption. Its unique anatomical characteristics predispose it to infection under certain conditions, which can subsequently progress to osteomyelitis. The blood supply to the pubic symphysis is derived primarily from branches of the obturator and internal pudendal arteries. Several factors contribute to an increased susceptibility to infection in pediatric patients, including the relatively small and less-developed collateral circulation, lower penetration of systemic antibiotics, and the high metabolic activity of the periosteum. Consequently, infections can readily disseminate bilaterally through Sharpey's fibers, extending along the pubic rami and potentially leading to extensive osteomyelitis or even abnormal fistulous connections with the pelvic or intra-abdominal organs. In the present case, the patient presented with restricted bilateral lower limb mobility, limping, and exacerbated pain with hip motion. The intraoperative findings confirmed infection of the pubic symphysis with bilateral pubic osteomyelitis, aligning with the above anatomical and pathological characteristics.

Infection of the pubic symphysis is a rare orthopedic infectious disease, and its diagnosis and management remain subjects of considerable debate. There is considerable inconsistency in the nomenclature used in earlier studies, with terms such as “pubic osteitis”, “pubic osteomyelitis”, “pelvic osteomyelitis”, and “septic arthritis of the pubic symphysis” often used interchangeably. The lack of a standardized designation complicates both clinical research and data integration, particularly in the differentiation of primary pubic symphysis infection from infections involving adjacent pubic structures. Based on a thorough literature review, we propose that “pubic symphysis infection” is the most appropriate terminology, as it provides an accurate delineation of the infection site within the symphyseal region. In terms of etiology, the pathogenesis of pubic symphysis infection is closely linked to its local anatomical characteristics. In adults, infection is frequently secondary to pelvic surgery, obstetric trauma, intravenous drug abuse, or intense physical activity, all of which can increase the risk of tissue injury and subsequent infection. Obstetric trauma, for instance, may lead to symphyseal diastasis, predisposing the region to infection. Similarly, intravenous drug users, due to compromised immune function and repeated local injections, are at an increased risk of infection. Additionally, repetitive high-impact physical activity can lead to microtrauma of the pubic symphysis, creating a susceptible environment for bacterial invasion. In pediatric patients, however, the epidemiology of pubic symphysis infection exhibits distinct characteristics. Studies indicate that there is significant variation in the width and maturation of the pubic symphysis according to age and sex, and the spectrum of bacteremia-causing pathogens differs between children and adults. Factors such as immune system immaturity may also contribute to these differences. In the present case, the patient had no identifiable high-risk factors, no history of pelvic surgery, and had not taken part in strenuous physical activity, reinforcing the notion that pediatric cases follow a different epidemiological pattern compared to adult cases. Therefore, when diagnosing and treating pediatric pubic symphysis infections, it is essential to consider these unique epidemiological and anatomical distinctions.

Infection of the pubic symphysis is a rare but serious condition, with early symptoms often being nonspecific and easily overlooked. Patients typically present with pain in the pubic symphysis region, which worsens with movement of the hip joint, restricting mobility and leading to limping. However, these clinical manifestations lack specificity and can be easily confused with other postpartum or postoperative pelvic conditions, such as pubic symphysis diastasis, non-infectious osteitis pubis, pubic symphysis injury, and pubovesical fistula. Additionally, especially in the early stages of the disease, significant bone destruction may not be visible on imaging examinations, further complicating diagnosis. MRI is the imaging modality of choice for diagnosing pubic symphysis infection. Its high-resolution soft tissue imaging capabilities enable the early detection of inflammatory changes, such as bone marrow edema, widening of joint spaces, and inflammatory infiltration of the surrounding soft tissue. In cases where infection is suspected, microbiological tests (e.g., blood or pus cultures) should be performed to identify the causative pathogens. Early MRI evaluation to distinguish infectious from non-infectious lesions can prevent disease progression, including sequestrum formation, abscess development, fistula formation, bilateral pubic ramus bone destruction, and loss of pelvic stability (11). In the present case, the patient's x-ray examination was unremarkable, whereas MRI revealed the presence of bilateral pubic osteomyelitis and an abscess in the posterior muscle layer of the pubic symphysis, further supporting the anatomical basis for the spread of infection in this region. Therefore, in high-risk patients, such as those with fever, and significantly elevated CRP levels and ESR, MRI should be performed even if initial x-ray findings appear normal to avoid misdiagnosis and delayed treatment. In terms of pathogen classification, pubic symphysis infections can be categorized into three types, namely, non-specific (e.g., *Staphylococcus aureus*), specific (e.g., *Mycobacterium tuberculosis*), and mixed bacterial infections. *Mycobacterium tuberculosis* is the most common pathogen responsible for specific bacterial infections and diagnosis is based on its isolation from pus or biopsy tissue. The primary treatment consists of anti-tubercular therapy, with surgical intervention considered in cases of extensive sequestrum formation, fistula development, and failure of conservative management, with generally favorable outcomes (12). Non-specific bacterial infections show similar presentations to non-infectious osteitis pubis, with the key distinguishing feature being an acute onset with fever. Common pathogens include *Staphylococcus aureus, Pseudomonas aeruginosa, Escherichia coli, Salmonella* species, and anaerobes. However, standardized diagnostic and treatment guidelines for these infections are lacking (13). The primary treatment approach involves pathogen-specific antibiotic therapy, with surgical intervention considered in selected cases. However, there is no consensus on the optimal timing for surgery, and the surgical techniques used vary widely. Common surgical approaches include thorough debridement, local placement of antibiotic beads, holmium laser debridement, or fixation of the pubic symphysis in refractory cases (14–17).

In this pediatric case, the causative pathogen was identified as methicillin-resistant Staphylococcus aureus (MRSA). Despite administering two weeks of intravenous broad-spectrum antibiotic therapy, the infection progressed locally, culminating in abscess formation. This outcome indicates the inadequacy of systemic administration in controlling the infection site. MRSA is known for its substantial biofilm-forming capacity, which allows it to evade host immune defenses and resist antibiotic treatment, thereby persisting at the infection site and complicating treatment while increasing the risk of recurrence (18). Although systemic antibiotics offer convenience, their effectiveness is contingent upon achieving sufficient drug concentrations at the site of infection. Penetration of antibiotics into bone tissue is often limited, particularly in regions with poor vascular supply, frequently resulting in subtherapeutic levels. This limitation was clearly evidenced by the progression to abscess formation in this case (19). Moreover, considering the limited vascularity of the pubic symphysis region and the lack of adjacent musculocutaneous flap coverage in this child, it was determined that local antibiotic delivery was crucial to attain significantly elevated drug concentrations within the infected focus. In the management of osteomyelitis caused by methicillin-resistant Staphylococcus aureus (MRSA), a prevalent approach involves comprehensive intraoperative debridement coupled with high-pressure pulsatile lavage exceeding 300 mmHg, alongside the implantation of a local antibiotic-loaded carrier. The pulsatile lavage serves to mechanically disrupt biofilms and improve tissue penetration. Concurrently, local antibiotic carriers facilitate sustained drug release, suppress residual bacterial proliferation, and diminish both the duration and dosage of systemic antibiotics, thereby mitigating the risk of developing antimicrobial resistance (20). In selecting an appropriate local delivery system, we considered the following options: Continuous local antibiotic perfusion (CLAP) facilitates the delivery of high concentrations of antibiotics directly to the site of infection, thereby maintaining sustained local drug levels that are effective in controlling pathogens. Nonetheless, parameters such as infusion concentration, flow rate, and treatment duration lack standardization, which can result in incomplete therapy. For example, acute osteomyelitis may necessitate short-term, high-concentration infusions, whereas chronic infections might benefit from prolonged, low-dose continuous administration (21, 22). Moreover, CLAP depends on catheter delivery, which poses risks of catheter-related infections and local complications (23, 24), as well as challenges such as significant wastage of costly antibiotics, catheter blockage, and complex nursing requirements. Consequently, this method was not adopted.Antibiotic-loaded beads have become increasingly prevalent in the treatment of orthopedic infections owing to their proven effectiveness. This study undertakes a comparative analysis of conventional polymethyl methacrylate (PMMA) beads and vancomycin-loaded calcium sulfate (VCS) beads.Polymethyl methacrylate (PMMA) as an antibiotic carrier demonstrates favorable mechanical properties and biocompatibility, rendering it appropriate for temporary structural support in weight-bearing regions. The release of vancomycin from PMMA follows time-dependent kinetics, characterized by an initial high release rate and elevated local concentration within the initial few days, subsequently declining gradually over a period of approximately 4 to 6 weeks (25). Nonetheless, PMMA is non-biodegradable and remains *in situ* following drug elution, necessitating a secondary surgical procedure for removal. Additionally, it is associated with incomplete antibiotic release and a tendency to facilitate biofilm formation, which may hinder the complete eradication of infection and impede bone healing.In contrast, VCS presents several advantages as an antibiotic carrier. Firstly, it is a bioabsorbable material, which obviates the necessity for secondary surgical intervention. Its porous architecture not only facilitates vascular ingrowth and osseointegration but also enhances the comprehensive release of antibiotics. Empirical studies have demonstrated that local concentrations of vancomycin achieved with VCS can be up to tenfold higher than those attained with PMMA, maintaining levels above the minimum inhibitory concentration (MIC) for a duration of 6 to 8 weeks (26). Furthermore, the degradation products of VCS, namely calcium and sulfate ions, possess osteoinductive properties that contribute to the repair of bone defects, thereby mitigating the risks associated with foreign body retention and secondary infection.Based on this comparative analysis, and given that the affected area in this instance was non-weight-bearing, vancomycin-loaded calcium sulfate (VCS) was chosen as the local sustained-release carrier. The surgical procedure involved intraoperative high-pressure pulsatile lavage, followed by meticulous debridement and the implantation of VCS. The patient's incision achieved primary intention healing within one week, accompanied by complete pain resolution and the restoration of normal activity levels. Subsequent follow-up assessments confirmed the eradication of the infection, satisfactory bone healing, and the absence of recurrence or systemic adverse effects. These findings underscore the significant advantages of VCS in managing osteomyelitis in hypovascular regions. Its capacity to deliver high local concentrations of antibiotics effectively addresses the pharmacokinetic limitations associated with systemic therapy, while its osteoinductive properties facilitate structural reconstruction. Consequently, VCS introduces a new treatment paradigm for recalcitrant infections by providing high-concentration local antibiotic therapy and obviating the need for implant removal, thereby eliminating the risks associated with retained foreign material (27).

## Conclusion

In summary, as shown by the present findings and literature review, pyogenic infection of the pubic symphysis can have an insidious onset. Due to its rarity, clinical awareness remains limited, often leading to significant delays between the time of symptom onset and definitive diagnosis. In the differential diagnosis, pyogenic arthritis of the pubic symphysis should be considered, particularly in pediatric cases presenting with groin or hip pain exacerbated by ambulation and accompanied by fever. Notably, the absence of fever does not rule out the diagnosis. Targeted clinical examination may reveal highly localized tenderness at the pubic symphysis. When suspected, MRI should be performed promptly. Definitive diagnosis relies on blood cultures, percutaneous aspiration cultures, and histopathological analysis. The use of vancomycin-loaded calcium sulfate bone grafts represents an effective treatment option for pediatric pubic symphysis infections. This study has several limitations. The small number of clinical cases of pubic symphysis infection (PSI) has resulted in a limited sample size and relatively short follow-up period, which may introduce bias in some outcome measures. In future research, we plan to extend the follow-up duration to obtain more comprehensive long-term efficacy and safety outcomes. Furthermore, we intend to employ vancomycin-loaded calcium sulfate (VCS) in eligible cases to accumulate additional clinical data. Concurrently, we will closely monitor local drug concentrations and systemic serum levels following VCS implantation, so as to provide a more robust evidence-based foundation for the standardized application of VCS in the treatment of refractory osteomyelitis in children.

## Data Availability

The original contributions presented in the study are included in the article/Supplementary Material, further inquiries can be directed to the corresponding author.
